# Structural analysis on mutation residues and interfacial water molecules for human TIM disease understanding

**DOI:** 10.1186/1471-2105-14-S16-S11

**Published:** 2013-10-22

**Authors:** Zhenhua Li, Ying He, Qian Liu, Liang Zhao, Limsoon Wong, Chee Keong Kwoh, Hung Nguyen, Jinyan Li

**Affiliations:** 1 School of Computer Engineering, Nanyang Technological University, Singapore 639798; 2 Advanced Analytics Institute and Center for Health Technologies, Faculty of Engineering and Information Technology, University of Technology Sydney, PO Box 123, NSW 2007, Australia; 3 School of Computing, National University of Singapore, Singapore 117417

## Abstract

**Background:**

Human triosephosphate isomerase (HsTIM) deficiency is a genetic disease caused often by the pathogenic mutation E104D. This mutation, located at the side of an abnormally large cluster of water in the inter-subunit interface, reduces the thermostability of the enzyme. Why and how these water molecules are directly related to the excessive thermolability of the mutant have not been investigated in structural biology.

**Results:**

This work compares the structure of the E104D mutant with its wild type counterparts. It is found that the water topology in the dimer interface of HsTIM is atypical, having a "wet-core-dry-rim" distribution with 16 water molecules tightly packed in a small deep region surrounded by 22 residues including GLU104. These water molecules are co-conserved with their surrounding residues in non-archaeal TIMs (dimers) but not conserved across archaeal TIMs (tetramers), indicating their importance in preserving the overall quaternary structure. As the structural permutation induced by the mutation is not significant, we hypothesize that the excessive thermolability of the E104D mutant is attributed to the easy propagation of atoms' flexibility from the surface into the core via the large cluster of water. It is indeed found that the B factor increment in the wet region is higher than other regions, and, more importantly, the B factor increment in the wet region is maintained in the deeply buried core. Molecular dynamics simulations revealed that for the mutant structure at normal temperature, a clear increase of the root-mean-square deviation is observed for the wet region contacting with the large cluster of interfacial water. Such increase is not observed for other interfacial regions or the whole protein. This clearly suggests that, in the E104D mutant, the large water cluster is responsible for the subunit interface flexibility and overall thermolability, and it ultimately leads to the deficiency of this enzyme.

**Conclusions:**

Our study reveals that a large cluster of water buried in protein interfaces is fragile and high-maintenance, closely related to the structure, function and evolution of the whole protein.

## Introduction

Human triosephosphate isomerase (TIM) deficiency, first reported by Schneider *et al. *in 1965 [1], is a genetic disease caused by the dysfunction of TIM. Clinical phenotypic characteristics of this disease include chronic hemolytic anemia and progressive neuromuscular disorder, which can eventually lead to early childhood death. In aged people, induced dysfunction of TIM is related to the neurodegenerative Alzheimer's disease [2,3]. Diseased cells of patients with TIM deficiency usually exhibit reduced TIM activity and a high level of the substrate DHAP. There are also many misfolded TIM proteins in diseased cells which accumulate to form large protein aggregates directly responsible for the neurodegenerative disorder [4]. A more recent study shows that reduced activity of TIM can lead to an oxidized redox state, making the subject sensitive to oxidative stress, and this explains why the disease phenotype progresses gradually [5].

More than 10 mutations [6] have been observed in *Homo sapiens *TIM (HsTIM) that causes the TIM deficiency, such as E104D [7], V231M [8], F240L [9], C41Y [10], and I170V [10]. The mutation E104D, a glutamate at position 104 substituted by an aspartate, occurs most often of these mutations. E104D was first studied by Daar *et al. *in 1986 [7] by comparing two unrelated TIM patients. An important finding is that the E104D mutant is thermolabile--that is, when the temperature goes up slightly, the enzyme is easily subject to destruction or great change, and then the enzyme loses its activity quickly. Following these pioneering studies [1,7], many other patients with the E104D mutation from several families across the world have been investigated [8,11-13]. All these patients are believed to originate from a common ancestor dated back to more than 1000 years ago [14]. In 1994, a recombinant HsTIM structure was solved at 2.8 Å resolution [15], and the local residue organization of GLU104 was unveiled. However, the conclusion--the E104D mutation changes the structure of the active sites through a chain of perturbations--is not exactly correct. Rather, in 2006, Ralser *et al. *concluded that TIM deficiency is caused by the altered dimerization but not the inactivity of the enzyme [16]. In 2008, the structure of E104D HsTIM mutant was solved at a better resolution 1.85 Å [17]. Comparing with the wild type structure, it was found that there is no significant change in the active site region, and found that the catalytic activity of the mutant enzyme is at the same magnitude as the native enzyme. However, the structural comparison of the mutated site between the wild type and the mutant reveals that there is a perturbation in the organization of the interfacial water molecules. Despite of these conflicting statements in the past research, the common understanding on the pathogenesis of the mutation E104D shares the following points: (i) There is an alteration of the binding of the two subunits in the E104D mutant [16]; (ii) The altered binding of the two subunits harms thermostability of the protein [1]; and (iii) the excessive thermolability causes the dysfunction of the enzyme [1]. These are wet-lab results and are hence valid. The only gap yet to be filled in the chain of knowledge explaining the pathogenesis of the mutation E104D is to find out how exactly the E104D alters the binding of the two subunits. It is already known that the interfacial water molecules play an important role in the binding of the two subunits [17]. However, the literature work does not answer why and how the interfacial water molecules contribute to the excessive thermolability of the mutant. In fact, it is unknown whether or not the presence of the large water cluster is responsible for the thermolability.

In this work, we conduct a comparative analysis on the wild type HsTIM and the E104D mutant's structures to understand the pathogenesis of this mutation and address three specific questions:

• why the mutation E104D can alter the binding affinity of the two subunits,

• how the mutation E104D introduces excessive thermolability to the enzyme, and

• what role the interfacial water molecules play.

**Figure 1:**
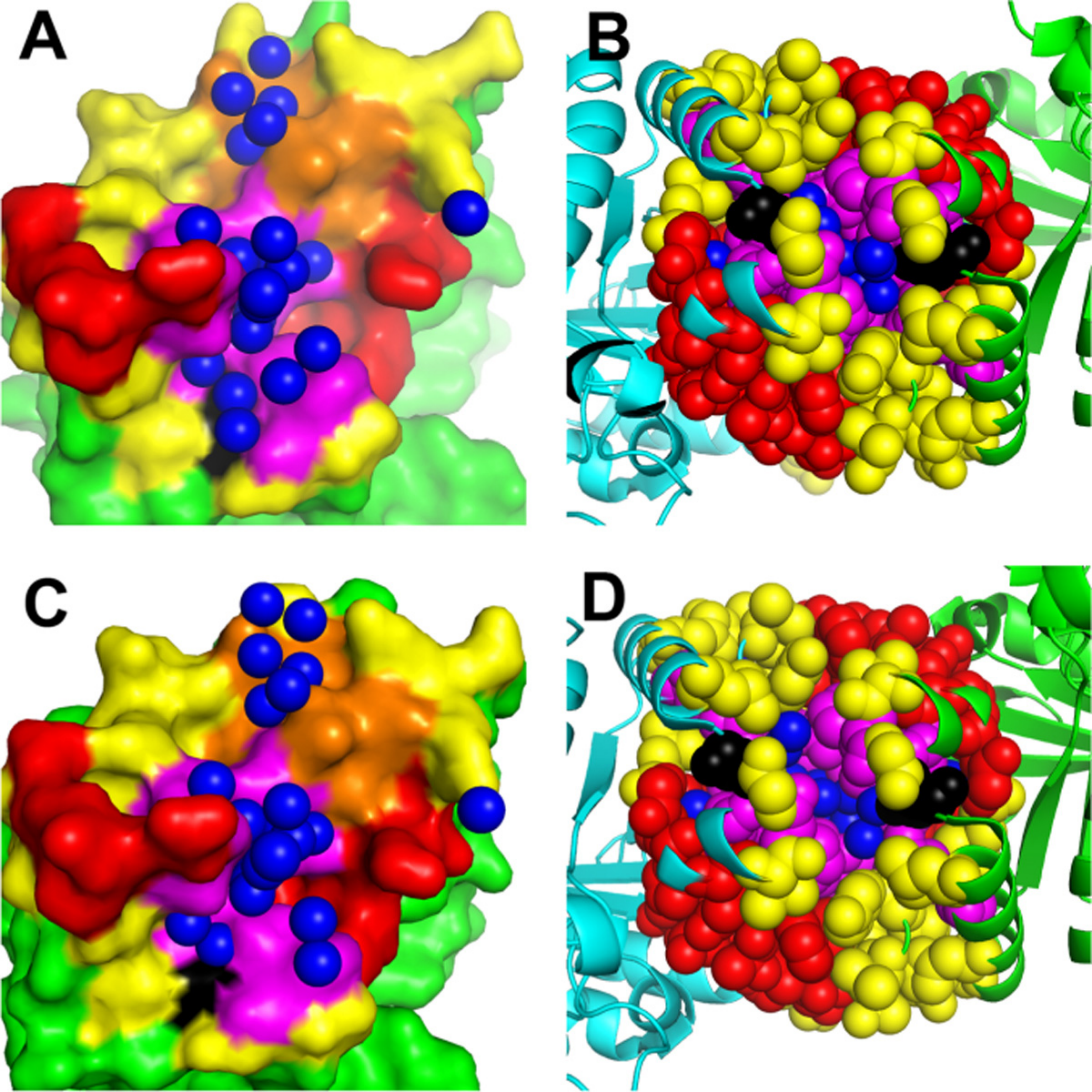
**Water distribution patterns in HsTIM wild type and E104D mutant dimer interfaces**. The water molecules in this interface are shown in blue spheres, and the non-interface region is in green. The interface are divided into four regions: regions A, B, C and D, and they are outlined by color magenta, orange, red and yellow, respectively. Subfigures (A) and (C) show one side of the wild type and mutant interface (in surface), respectively, and (B) and (D) show side views of the wild type and mutant interface, respectively. The residue GLU104 (in (A) and (B)), and ASP104 (in (C) and (D)), which are in region A, are highlighted in black.

The HsTIM dimer interface is a very abnormal interface in terms of the distribution of water molecules. The water molecules are unevenly distributed in an atypical "wet-core-dry-rim" manner. Most of the 25 interfacial water molecules are tightly enveloped by 22 residues in a deep region (denoted by region A) with the residue GLU104 at the periphery (Figure 1). Both the water and the residues in region A are well conserved in eukaryotic and bacterial TIMs (dimers), but the conservation is not observed in archaeal TIMs (tetramers). These facts motivate us to examine whether this large cluster of water in region A is needed in nature to maintain the quaternary structure of non-archaeal TIMs, and whether the protecting residues have to be conserved as well to maintain this water cluster during evolution. The overall dimer interface of E104D HsTIM mutant has a similar size and wetness as that of the native enzyme, and the structural perturbation in terms of the spatial movement of atoms in this mutant is not significant. The native and the mutant also have the same number of interfacial water molecules. All these indicate that the pathogenesis of the mutation is not simply attributed to the structural perturbation of the interface.

This work goes beyond the scope of structural perturbation studies. We hypothesize that the large cluster of water in region A transmits the atoms' mobility from the surface deeply into the core of the interface, and that this mobility transmission can harm the thermostability of the subunit binding and the function of the protein. As the B factor of atoms is closely related to the thermolability of a protein [18,19], the elevated normalized B factors are used to understand the increased flexibility of the interfacial water and other atoms clustered near the mutated site (i.e., water in region A).

To validate our hypothesis of the role of water cluster in region A, molecular dynamics simulations were run for the wild type and the mutant HsTIM. The difference in the root-mean-square deviation (RMSD) between the two structures over the simulation is only observed in region A. For other interfacial regions and the whole protein, the RMSD does not change much. Thus we conclude that it is the water molecules in region A that are directly responsible for the excessive thermolability of the enzyme.

As highlights, our pathogenesis study for the mutation E104D is to affirm: (i) this mutation can lead to an excessive thermolability of the enzyme, and (ii) the binding between the two subunits is harmed by the increased flexibility of the interfacial atoms, which are amplified by the large cluster of buried water near the mutation site. In other words, the abnormal organizational topology of water molecules in the HsTIM dimer interface is the key to understanding the fundamental mechanism of the HsTIM deficiency. Our results can be generalized to understand that large clusters of water molecules in protein binding interface are very high-maintenance, and they can greatly affect the overall structure, function and evolution of the protein.

## Methods

### Protein structures used in this study

The structures of wild type HsTIM and mutant E104D are taken from a previous study [17]. To get the TIM structures of other species, a search of "triosephosphate isomerase" in the protein data bank was performed. And the structures are selected manually. TIM structures collected by Wierenga *et al. *[20] are also considered. After removing some structures with mutations, or without solvent information, we get the wild type TIM structures from 20 different species. The detailed information of these structures can be found in Table 1.

**Table 1:** TIM structures and subunit interfaces used in this study.

Domain	PDB	Organism	Resolution (Å)	#Water	#Atoms	Wetness	rWBL	gini
Eukarya	2JK2	*H. sapiens*	1.70	25	546	0.046	1.282	0.684
	1R2R	*O. cuniculus*	1.50	30	563	0.053	1.140	0.681
	1TPH	*G. gallus*	1.80	23	517	0.044	1.270	0.724
	2I9E	*T. molitor*	2.00	17	498	0.034	0.894	0.687
	1MO0	*C. elegans*	1.70	29	530	0.055	1.063	0.620
	1YPI	*S. cerevisiae*	1.90	22	503	0.044	1.304	0.649
	1TPF	*T. brucei*	1.80	22	479	0.046	1.034	0.633
	1TCD	*T. cruzi*	1.83	19	464	0.041	1.004	0.655
	1AMK	*L. mexicana*	1.83	23	483	0.048	1.017	0.630
	1M6J	*E. histolytica*	1.50	23	490	0.047	1.159	0.631
	1YDV	*P. falciparum*	2.20	28	540	0.052	1.049	0.515

Bacteria	1TRE	*E. coli*	2.60	12	491	0.024	1.293	0.671
	1AW2	*M. marina*	2.65	17	478	0.036	1.032	0.709
	1B9B	*T. maritima*	2.85	11	502	0.022	1.162	0.680
	3GVG	*M. tuberculosis*	1.55	41	587	0.070	1.132	0.594
	3KXQ	*B. henselae*	1.60	46	600	0.077	1.121	0.502
	2JGQ	*H. pylori*	2.30	31	555	0.056	1.146	0.525

Archaea	1HG3	*P. woesei*	2.70	18	458	0.039	1.103	0.611
	2H6R	*M. jannaschii*	2.30	5	400	0.013	1.268	0.747
	1W0M	*T. tenax*	2.50	21	487	0.043	1.075	0.516

The TIM structures from 20 organisms used and their properties: resolution, number of interfacial waters, number of interfacial atoms, wetness, rWBL and gini coefficient (from column 4 to column 9).

The water distribution topology of HsTIM dimer interface is compared with a data set of homodimeric interfaces. They are selected from 206 obligate interfaces collected from a few previous studies [21-24]. Obligate protein interfaces are considered as a homodimeric interface if the two interacting protein chains are identical. Some interfaces with less than 10 water molecules are removed as their rWBL and gini coefficient (defined later) are of large variance. Finally, 91 homodimers are left and they are used in a comparative analysis to illustrate the abnormality of HsTIM dimer interface. The information of these 91 interfaces and their properties are available in Additional file 1.

### Definitions of protein-water-protein tripartite interface, burial level and gini coefficient

We consider only heavy atoms in this study. Water molecules immobilized in a protein/protein complex are considered as a part of the protein/protein complex rather than a part of bulk solvent. Water molecules with a larger than 10 Å^2 ^solvent accessible surface area (SASA) are defined as exposed as a part of bulk solvent and they are removed iteratively from a structure until no water molecules are exposed in the structure. The remaining structure including those buried water molecules are considered as the protein complex and are used in the subsequent processes in our work.

For each protein complex structure, an atomic contact graph is defined with atoms as its nodes and atomic contacts as its edges. Atomic contacts are defined by combining the Voronoi diagram and an atom-atom distance. The distance threshold for an atomic contact between two atoms is the sum of their radii plus the diameter of a water molecule 2.75 Å.

#### Protein-water-protein tripartite interface

We define the interface between two proteins as a tripartite graph, which is a subgraph of the atomic contact graph of the whole protein complex. The nodes of an interface tripartite graph consist of three sets of atoms: the oxygen atoms of the interfacial water molecules and the atoms from the two interacting partners that have atomic contact with interfacial water or with the interacting partner. A water molecule is defined as interfacial water if it has atomic contacts with both sides. The edges in the tripartite graph are the atomic contacts among the three sets. In this way the atom level interface is defined as a set of atoms. We also define any residue that has at least one atom in the interface as an interfacial residue.

#### Burial level and relative water burial level

The burial level of an atom *a *(denoted as *BL_a_*) in a protein complex is calculated in the atomic contact graph. It is the length of the shortest path to the nearest exposed atom-an atom with an SASA larger than 10 Å^2^.

The relative water burial level (rWBL) is calculated as the average burial level of water divided by the average burial level of all interfacial atoms:

(1) $$rWBL=\frac{\sum_{a\;\in I_{W}}BL_{a}/|I_{W}|}{\sum_{a\;\in I}BL_{a}/|I|}$$

where *I *is the set of atoms in the interface, *I_W _*is the oxygen atoms of the water molecules in interface, and *|I| *is the cardinality of set *I*, which is the number of atoms in this interface. According to this definition, a large rWBL indicates that the water molecules are deeply buried.

#### Gini coefficient of water distribution

Gini coefficient is widely used to describe the unevenness of a distribution. Usually it is used to measure the inequality of income or wealth. Its value is between 0 and 1. A lower gini coefficient indicates a more uneven income distribution of a country. Gini coefficient is defined based on the Lorenz curve [25]. Lorenz curve is a plot of the cumulative share of population ordered from the lowest income to the highest income versus the cumulative share of the income. If the incomes are absolutely equal, the Lorenz curve will be a straight line, which is called line of equality. The gini coefficient is defined as the area between the Lorenz curve and the line of equality divided by the area under the line of equality.

Here, we borrow the concept of gini coefficient to address how unevenly the water is distributed in an interface. The (probabilistic) distribution of the number of contacting interfacial water molecules (of interfacial non-water atoms) is used. In our case, for an interface, the Lorenz curve is the connection of several successive line segments, where the horizontal axis is between 0 and *n *(*n *is the number of non water interfacial atoms) and the vertical axis between 0 and $\sum_{j=0}^{n}y_{j}$. Here, *y_j _*is the number of contacting interfacial water molecules of interfacial atom *j *(*y*_0 _is set to 0) and the sequence from *y*_1 _to *y_n _*is sorted in a non decreasing order with *y*_*j *_≤ *y*_*j*+1_. The Lorenz curve starts at point (0, 0), and then a line segment is created between each (*i - *1, $\sum_{j=0}^{i-1}y_{j}$) and (*i*, $\sum_{j=0}^{i}y_{j}$). Let *X *be the area between the Lorenz curve and the line of equality (i.e. the line segment between (0, 0) and (*n*, $\sum_{j=0}^{n}y_{j}$)), and let *Y *be the area under the line of equality. The gini coefficient of the interface is defined as:

(2) $$G=\frac{X}{Y}=\frac{\sum_{i=1}^{n}iy_{i}}{n\sum_{i=1}^{n}y_{i}}+\frac{n+1}{n}$$

### Structural alignment, water correspondence and B factor normalization

As the sequences of the wild type HsTIM and E104D mutant differ only from each other in one position, we perform structural alignment of the two structures by superimposing them using Pymol [26]. The algorithm is based on the amino acid sequences of the two structures. Once the aligned wild type and mutant structures are obtained, the 25 water molecules in wild type are searched in the mutant structure to determine whether it reappears or not. If two interfacial water molecules, one in wild type and the other in mutant, are within 1.0 Å distance from each other, and they are mutually the closest water molecule to each other, we say the water in the wild type structure reappears in the mutant.

Due to the environmental differences when a protein structure is solved, the B factors in different structures cannot be compared directly. The B factor of a protein complex is normalized within the complex as:

(3) $$B_{*}=\frac{B-{\bar{B}}_{a}}{\sigma_{a}}$$

where *B *is the original B factor reported in the PDB record, and ${\bar{B}}_{a}$ and *σ_a _*are the mean and standard deviation of the B factors of non water atoms which are at least 15 Å away from the *C^γ ^*of the mutated residue--GLU104. We do not consider water here because the water information quality is correlated with the resolution [27] and, maybe, other environmental issues. We also exclude the atoms near the mutated site from calculating mean and standard deviation as the mutation is expected to change the B factors of atoms near it. In this way we compare the B factor of an atom in wild type and mutant with non water atoms far from the mutation site as references.

### Molecular dynamics simulation

Two molecular dynamics simulations were run for the wild type [PDB: 2JK2] and the E104D mutant [PDB: 2VOM] HsTIMs. Buried water molecules were included in the initial structures. For both simulations, the protein structure was solvated in a water box where a minimum of 10 Å distance was kept between the protein and the boundary. Charges were neutralized by either Cl^- ^or Na^+ ^ions. Solvation and ionization were performed using the the VMD software [28].

For both simulations, CHARMM22 parameter set [29] with CMAP correction [30] was used, and the step size was set to 2 fs. The solvated and ionized system was minimized for 1000 steps and simulated for 2500000 steps (5 ns). Initial velocities were set with human body temperature (310 K). Langevin piston pressure control was used to control the system pressure at 1 atm. Periodic boundary conditions were used, and a threshold cutoff of 12 Å was set for non-bonded interactions. Particle mesh Ewald method [31] was used to calculate long-range electrostatic interactions. The simulations was carried out with the NAMD software [32].

## Results

We first present results to illustrate how exactly atypical the HsTIM dimer interface is, followed by results on the evolution of the water and residues in the interface of TIMs. We then present a hypothesis to understand the pathogenesis of the mutation E104D, and validate the hypothesis by molecular dynamics simulations.

### The abnormal hydration in HsTIM dimer interfaces

Figure 1(A) shows one side of the interface and all of the 25 interfacial water molecules (in blue). We divide the interface residues and water molecules into four regions for detailed analysis: (i) region A which is a wet region consisting of 16 water molecules and their surrounding 11 residues (in magenta and black color) from each side including the pathogenic mutation site 104 (in black color), (ii) region B which is another wet region with 5 water molecules and 6 residues (in orange color) from each side, (iii) region C (in red color) which is the functional region of the enzyme, containing 15 residues from each side including three of the four active site residues (ASN11, LYS13 and HIS95), and (iv) region D (in yellow color) which is a region in the interface containing residues and water that are not in region A, B or C. As the interface is homodimeric and symmetric, the residues in these 4 regions can be doubled actually.

Specifically in region A, the residue GLU104, whose mutation into aspartate causes TIM deficiency, is highlighted in black as shown in Figures 1(A) and 1(B). It can be seen that this residue is located at the waterside and reaches its side chain deeply into the water cluster in region A to "lock" this large amount of water molecules inside.

The total 25 water molecules in this interface account for 4.6% of the entire interfacial atoms. This wetness is similar to the average wetness of obligate interfaces [33]. However, it can be seen that these water molecules are distributed in an unusual way with a very special topology. First, most of these water molecules are deeply buried and clustered near the core of the interface. Second, these water molecules are unevenly distributed, mainly in two wet regions. Normally, water molecules in protein binding interfaces are organized in a "dry-core-wet-rim" manner with most water molecules placed near the rim and the wetness goes down progressively from rim to core [33]. This interface does not follow such a trend at all. In particular, the average burial level of the water molecules is 2.32, yielding an extraordinarily high rWBL (the relative burial level of water in an interface, see Materials and Methods) of 1.282 with regard to the average burial level of all the interfacial atoms 1.81. This kind of large water cluster is also unusual in terms of binding free energy, as immobilizing water molecules at the core of a protein interface is energetically expensive [34].

**Figure 2:**
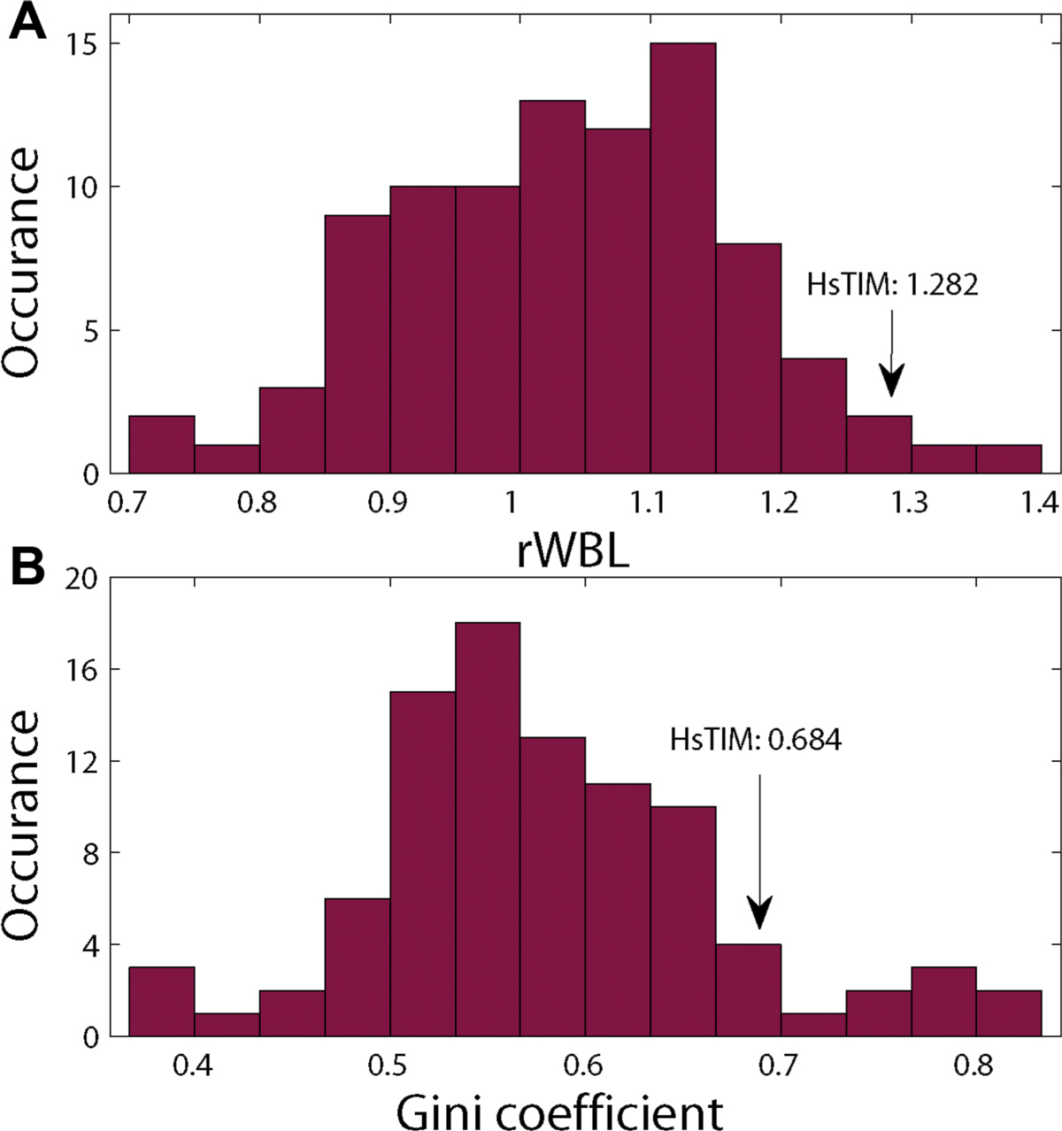
**The abnormality of HsTIM dimer interface**. Distribution of (A) the rWBLs and (B) the gini coefficients of the 91 homodimeric interfaces. The rWBL and gini coefficient of the HsTIM dimer interface are marked by arrows.

To see more about this water distribution abnormality, we compared the HsTIM dimer interface with 91 other homodimer interfaces by calculating their rWBL and gini coefficients. Results are shown in Figure 2. The rWBL describes the extent to which the water molecules are deeply buried and the gini coefficient indicates how uneven the water distribution in the interface is. Both the rWBL and the gini coefficient of HsTIM dimer interface are very high, comparing to the 91 homodimeric interfaces, indicating that the water distribution in this interface is very different from typical homodimeric interfaces. In Figure 2, a few interfaces have a higher rWBL or a higher gini coefficient than HsTIM dimer interface. Note that most of these interfaces are very dry with very few interfacial water molecules. When the number of interfacial water is small, the variance of rWBL is very large, and the gini coefficient is large due to fewer water-contacting atoms. The distribution pattern of water molecules in these interfaces is thus of low significance.

### Evolutionary studies on HsTIM dimer interface hydration

There are 21 species' wild type triosephosphate isomerase structures that have been solved by X-ray crystallography and the data is deposited in the protein data bank [35] as of 2011. One of them (PDB: 1BTM) does not have solvent information, so it is excluded from our analysis. The 20 TIM structures are listed in Table 1.

**Figure 3:**
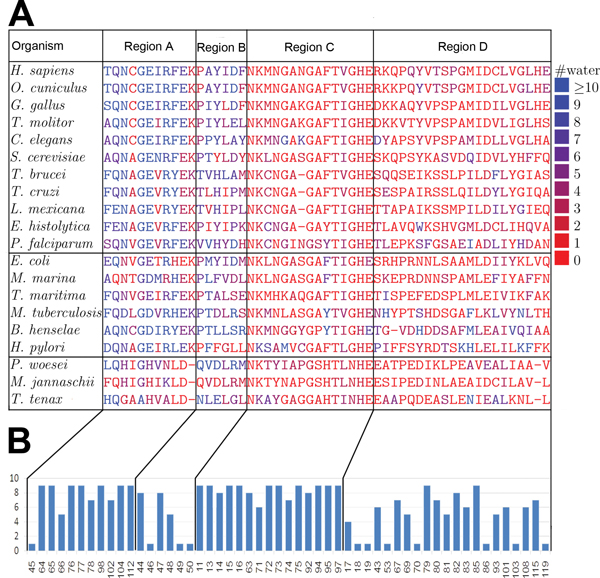
**Alignment and hydration profile of TIMs**. (A) Interfacial residues in sequence alignment (according to the HsTIM dimer interface) and their hydration profiles. The color of the the letters indicates the number of contacting interfacial water molecules of the residue (#water). The 20 interfaces are grouped into three categories: 11 Eukarya, 6 Bacteria, and 3 Archaea. (B) shows the corresponding conservation score of HsTIM dimer interface residues according to Consurf. Residues' positions in (A) and (B) are aligned and they are divided into four subregions according to HsTIM dimer interface.

**Figure 4:**
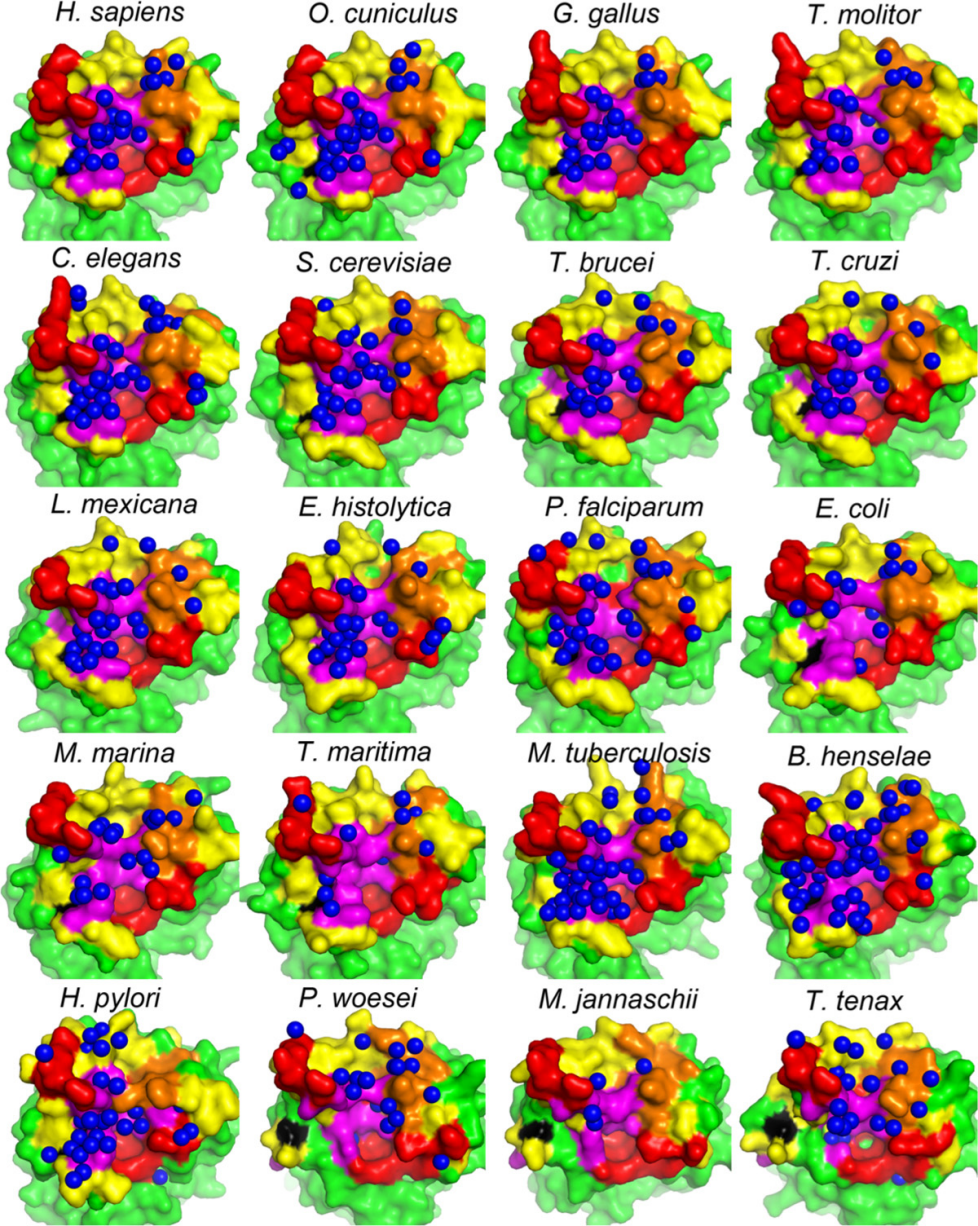
**Regions A, B, C and D in TIMs**. (A) Regions A, B, C and D in the TIMs of the 20 species in our data set. This figure shows the four regions in HsTIM dimer interface (the panel in the upper left corner) and their aligned residues in the TIMs from the other 19 organisms. Residues are colored with the same scheme as Figure 1. The water molecules (blue spheres) shown in each panel are the interfacial water. Please note that these figures are not the actual interfaces (except the one of Homo sapiens TIM), but just the part that are aligned with HsTIM dimer interface, though the water molecules are actual interfacial water.

Modern eukaryotic TIMs are believed to have the alpha-proteobacterial origin [36]. Thus, they are more similar to bacterial TIMs than to archaeal TIMs. Eukaryotic and bacterial TIMs are dimers and archaeal TIMs are tetramers. In Figure 3(A), multiple sequence alignments of the interface residues (according to HsTIM) and their interfacial hydration profiles are shown. In Figure 4, organization of interfacial water molecules and the four regions (aligned in sequence with the four regions in HsTIM) are shown. An archaeal TIM tetramer has two distinct inter-subunit chain-chain binding interfaces, and here the one with the larger overlap with HsTIM dimer interface is used. In Figure 3, the 20 organisms are ordered with the 11 eukaryotes at the top, the 6 bacteria in the middle and the 3 archaea at the bottom. The conservation according to Consurf [37] of these positions are shown in Figure 3(B), accordingly.

As shown in Figure 3(A), region A is wet in all of the eukaryotic and bacterial TIMs, indicating a large cluster of water is present in all non-archaeal TIMs. In Figure 4, it seems that three bacterial TIM dimer interfaces (in *E. coli*, *M. marina *and *T. maritima *TIMs) are dry in region A. However, these three structures are solved at very poor resolutions (Table 1), which usually implies a under-reporting of water information [27]. Meanwhile, as in the other non-archaeal TIMs, large inter-protein cavities are observed in these structures, which may accommodate water molecules. Thus, we believe these three interfaces are as wet as the other non-bacterial TIM dimer interfaces. Region A of the archaeal TIMs does not have as much water as that of the non-archaeal organisms, as can be seen from Figure 3(A) and Figure 4. Furthermore, three positions (positions 102, 104 and 112 according to HsTIM numbering) in the eukaryotic and bacterial TIM dimer interfaces are not in the corresponding interface in the archaeal TIMs. Positions 104 (where the E104D mutation occurs) and 112 are fully conserved in eukaryotic and bacterial organisms, always with position 104 as glutamate and position 112 as lysine. In the archaeal TIMs, position 104 changes to aspartate. Recall that, the E104D mutation in HsTIM, where the glutamate at position 104 is substituted by aspartate, is pathogenic. Also, as can be seen from Figure 4, in the archaeal TIMs (the last three panels), positions 102 (colored in magenta, left bottom) and 104 (in black) are separated from the other residues in region A (large area colored in magenta). Position 112 does not even exist in the archaeal TIMs.

These observations affirm that the large cluster of water in Region A is essential for the eukaryotic and bacterial TIMs. It contributes positively to the binding affinity, because if the interface loses the large cluster of water (as in the archaeal TIMs), the enzyme must adopt another quaternary structure to stabilize the enzyme. In evolution, the tertiary structures of non-archaeal TIMs are not ready to oligomerize into tetramers or other higher order oligomers. So, excluding such a large cluster of water from the interface without harming the function of the enzyme seems too hard--once a mutation disturbs the water topology in this region (such as E104D), it would significantly destabilize the enzyme and further turn off its functionality. Thus safely excluding these water molecules requires simultaneous mutation of many residues, which is too difficult in natural evolution. That is the reason why the large water cluster is necessary and is well maintained when TIMs are in the form of dimers. Also, to maintain such a large pocket of water, its nearby residues must be conserved.

Region B seems to be wet in almost all of the 20 TIMs. Region C's water contacting profile is not very conserved. In human it is dry, but in some other species, such as *C. elegans *and *H. Pylori*, it is wet. The hydration profile of region D is not very conserved, either. Combining these hydration profiles and conservation scores of the four regions, we can conclude that

• region A: both the water and residues are conserved in the bacterial and eukaryotic organisms;

• region B: only water is conserved;

• region C: only residues are conserved; and

• region D: neither water nor residue is conserved.

### Comparison between wild type and E104D mutant: why this mutation is pathogenic

The subunit interface of HsTIM E104D mutant is shown in Figures 1(C) and 1(D). It is very similar to the wild type interface as shown in Figures 1(A) and 1(B). Other comparison is shown in Table 2. We can see that the two interfaces have a similar interface size in terms of both ∆SASA (half of the solvent accessible surface area change upon binding [38]) and the number of interfacial atoms. The two interfaces also have the same number of water molecules. However, the water molecules at the mutated interface are buried shallower, as indicated by the lower rWBL. Nevertheless this rWBL is still high, compared with other homodimeric interfaces (Figure 2(A)).

**Table 2:** Comparison between HsTIM wild type and E104D mutant dimer interfaces.

Properties	∆SASA (Å^2^)	#Water	#Atoms	wetness	rWBL
Wild type	1800.6	25	546	0.046	1.28

E104D	1761.8	25	545	0.046	1.18

Overall comparison of the ∆*SASA*, #Water (number of interfacial water molecules), #Atoms (number of interfacial atoms), wetness and rWBL between HsTIM wild type and E104D mutant dimer interfaces.

#### The overall structural perturbation by the mutation is not significant

**Figure 5:**
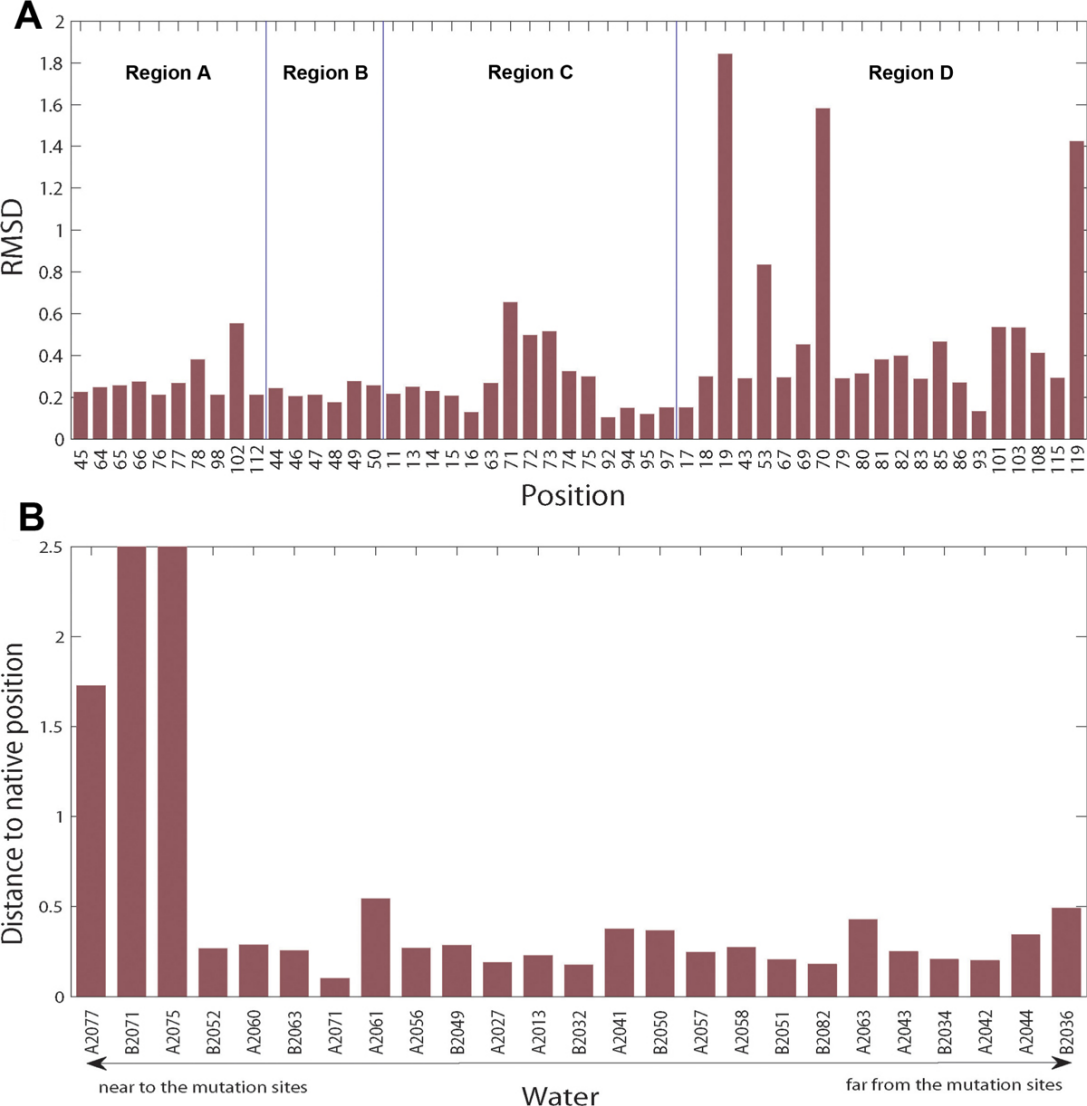
**Structural perturbation of the mutation**. (A) The RMSD of the interfacial residues and (B) the distance of the 25 water molecules in wild type interface to their nearest water in the mutant. The water molecules are sorted by their distance to the mutation sites. For water B2077, a water molecule in the mutant that is mutually the closest to it is observed with a distance larger than 1.0 Å (not reappear). For waters B2071 and A2075, no water that is mutually closest to them are observed, so a bar higher than 2.5 Å is shown for them. The remaining 22 waters reappear in the mutant.

The root-mean-square deviations (RMSDs) of the 53 interface residues in the structural aligment of the wild type and the E104D mutant are shown in Figure 5(A). A large RMSD indicates a large structural change. Only a few residues (positions 19, 70 and 119 in region D) have large position changes. Actually, the structural change of these residues is not due to the mutation but due to their solvent exposure. If we superimpose the two subunits of the wild type enzyme to each other, the RMSDs of residue 19 and 119 also have very large RMSDs as 1.32 Å and 1.28 Å, respectively. Residue 70's RMSD in the native structure is low at 0.29 Å, but this residue is highly exposed and the largest change in this residue is at the side chain, pointing out to bulk solvent from the enzyme. For those residues in regions A, B and C, the RMSDs are not very large. Specifically, in region A, where the mutated residue GLU104 is situated, most of the residues do not have a high RMSD even when they are very close to position 104. When the mutation E104D was first studied by Daar *et al. *[7], the mutation was thought to alter the active site through a chain of residues: 104 → 98 → 75 & 77 → 11 & 97 → 13, where residues 11 and 13 are active site residues in the interface. As can be seen from Figure 5(A), none of these residue has a large RMSD, indicating that the mutation does not alter the active site of the enzyme. This is also supported by the observation that the mutant enzyme has almost the same activity as wild type enzyme at normal temperature [17].

**Figure 6:**
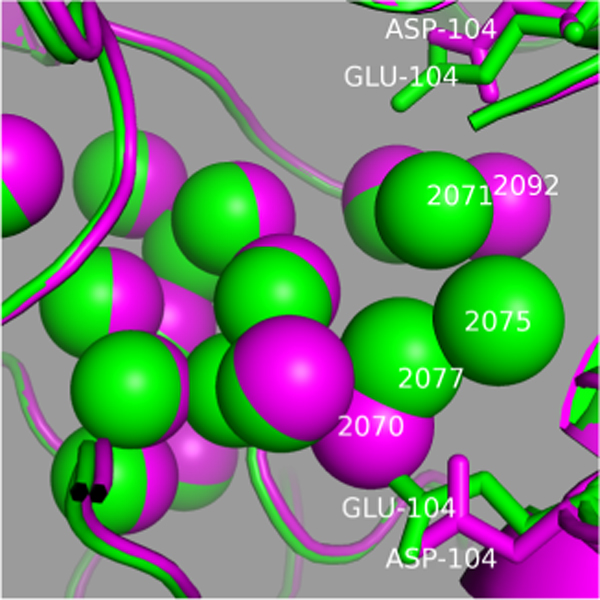
**Structural alignment between wild type E104D mutant HsTIM dimer interface**. Structural alignment between wild type (in green) and E104D mutant (in magenta) of region A of HsTIM dimer interface.

We also computed the distance to the nearest water in the mutant of the 25 water molecules in the wild type interface (Figure 5(B)). If two water molecules, one in wild type and the other in mutated enzyme, are the closest one of each other and their distance is less than 1.0 Å, we say the water (in wild type enzyme) reappears in the mutant. Twenty-two out of 25 water molecules reappear. All the 3 water molecules that do not reappear are in region A and they are very close to the mutation site-GLU104. Thus, the main body of the water cluster in region A is maintained. This can be observed in Figure 6. In this figure, the overall structure of the large water cluster in region A is not changed. Most water molecules in wild type enzyme have a corresponding water in the mutant, within a very small spatial distance.

#### Propagating atoms' mobility by water in region A: a hypothesis to explain the excessive thermolability of the mutant

As shown in the previous section, the overall structural change in the interfacial atoms and water molecules are not significant, and the overall structure of the interface is maintained in the E104D mutant. Thus the structural perturbation theories [7,15] cannot explain why the mutation is pathogenic. Recall that the mutation only makes the enzyme thermolabile, and the mutant is still functional at normal temperature. We thus hypothesize that the extra-large cluster of water molecules in region A propagates the flexibility of interfacial atoms deeply down to the core of the interface, adding excessive thermolability to the binding of the two subunits. As monomers of TIM is not functional, the thermolability of the TIM function is thus observed.

To get a better understanding of this hypothesis, we investigated the B factor of interfacial atoms in the two structures. The B factor of an atom indicates the level of thermal motion of the atom around its average position. It is closely related to the thermolability of a protein. It was found by Reetz's group that the thermostability/thermolability of a protein can be manipulated by using B factor as a guide [18,19]. Thus, B factor can also be used to study the thermostability/thermolability of the wild type and mutated HsTIM.

**Figure 7:**
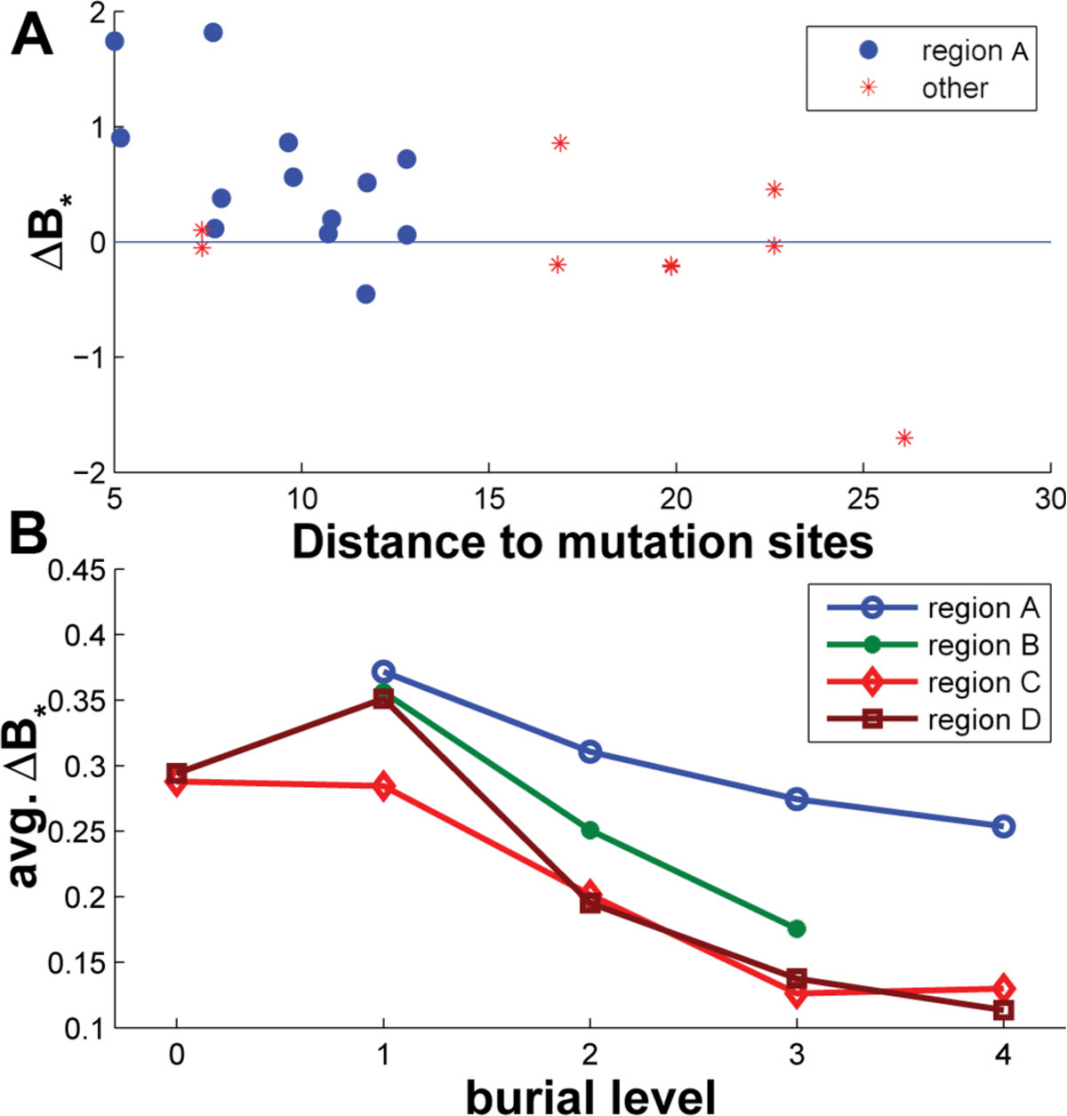
**Comparison of the normalized B factor in the wild type and E104D mutant structures**. (A) Normalized B factor change after the mutation E104D of the 22 reappearing interfacial water molecules in region A (blue, solid marker) and other regions (red, asterisk marker) versus the distance to the mutation sites, and (B) Average normalized B factor change of the interfacial atoms in regions A (blue, circle marker), B (green, solid marker), C (red, diamond marker) and D (brown, square marker) at different burial levels.

In Figure 7(A), a scatter plot of the change in normalized B factor after the mutation (∆B_*_) versus the distance to the mutation sites (*C^γ ^*of GLU104) of the 22 reappearing water molecules in the interface is shown. A negative correlation between them is observed, which is understandable as the nearer an atom is to the mutation, the more it will be affected by it. Most of the water molecules in region A have their normalized B factor increased, even when they are far from the mutation. Water molecules not in region A usually have a low normalized B factor change, including two water molecules that are very close to the mutation sites. These observations indicate that the mutation increases the mobility of the water molecules in region A, but this effect cannot propagate to the water molecules in the other regions, even when they are spatially close to the mutation. Thus, it is the large cluster of water in region A that enhances the mobility change.

In order to better understand the role of the large water cluster in region A in enhancing the interface mobility after the mutation, the average change in normalized B factor of interfacial non water atoms is plotted at different burial levels (the burial levels in the wild type enzyme); see Figure 7(B). First of all, the mobility of an interface atom tends to increase no matter where the atom is located or how deep it is buried, as all the average changes are larger than 0. Second, the mobility of atoms in region A increases the most comparing with the mobility increment of atoms in the other three regions. More importantly, the increase in mobility propagates itself deeply into the core of region A, while in other three regions the mobility increment drops quickly when burial level goes up. When the region has more water, the mobility increment decrease slower when the burial level goes higher, and that is why region B also have a higher mobility increment than the two dry regions (regions C and D), though it is much drier than region A.

The mobility change of the interfacial water molecules and atoms indicates that after the mutation, water and atoms in region A are more flexible, and the flexibility increment flows to those deeply buried atoms in this region through the large water cluster. This change is far weaker in other dry regions.

#### Molecular dynamics simulations: validating the role of interfacial water

**Figure 8:**
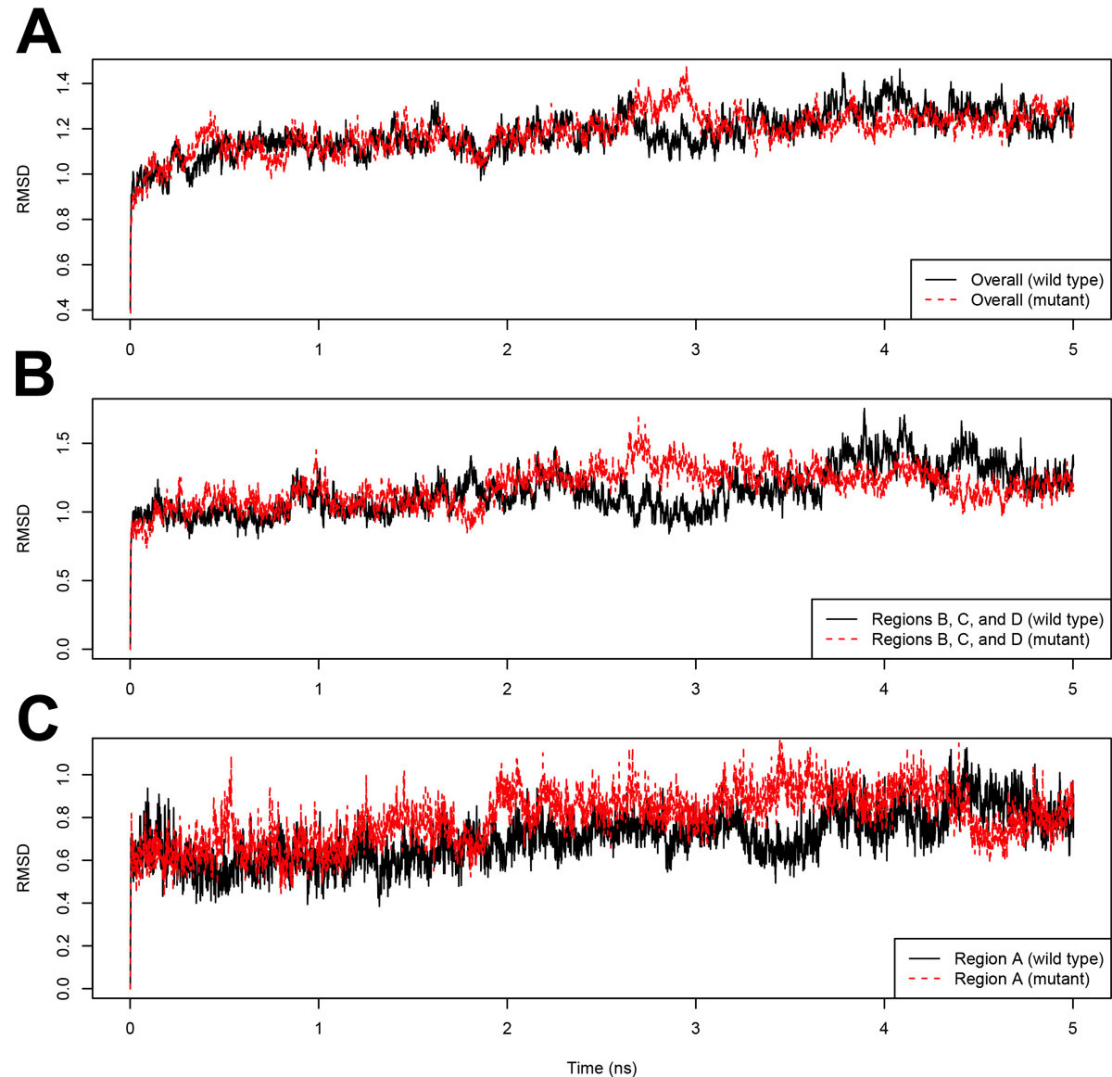
**Comparison of the RMSD of the wild type and E104D mutant structures in molecular dynamics simulations**. The RMSD of (A) the whole protein, (B) regions A, B and C in the subunit interfaces and (C) interfacial region A. RMSD (Å) values for each frame are calculated with the initial structures as the references. In all subfigures, the RMSD of the wild type structure is shown as black solid lines while the RMSD of the mutant structure is shown as red dashed lines.

To validate our hypothesis proposed in the previous section, molecular dynamics simulations were run for the wild type and E104D mutant HsTIMs at human body temperature (310 K) for 5 nanoseconds. Figure 8(A) shows the overall root-mean-square deviation (RMSD) of the two structures over the simulations using the initial structures as references. We can see that for both structures, the simulations reach equilibrium rapidly within 1 ns. Comparing the two simulations, the RMSD of the mutant structure is similar to that of the wild type protein, which is understandable as we know that the mutant is functional as the wild type at normal temperature. Thus overall the mutant is as stable as the wild type protein.

Regional RMSD of the subunit binding interfaces is compared in Figures 8(B) and 8(C). For interfacial regions B, C and D, the RMSD in the mutant is also similar to that in the wild type structure. Interestingly, a remarkable difference of RMSD is observed in region A, where the RMSD in the mutant is in general higher than that in the wild type, indicating that excessive flexibility is gained after the mutation. Meanwhile, at normal temperature, the excessive flexibility in region A does not reach other regions that is not in contact with the large cluster of water. This observation clearly shows that it is the large cluster of water that is responsible for the thermolability of the mutant as region A in the subunit interface is the only region that is more flexible after the mutation.

From the abovementioned analyses, we can conclude that the large cluster of water in region A is responsible for the excessive thermolability of the mutant. Although this work only analyzed the mutation E104D, the concept can be generalized to understand the high-maintenance of large water clusters in proteins. In HsTIM, we believe that this large cluster of water may also play important role in the pathogenesis of other point mutations in this enzyme and this water cluster could also be one of the reasons why there are so many pathogenic point mutations in this enzyme [6].

## Conclusion

An investigation of a mutation that causes HsTIM deficiency, E104D, is conducted. The HsTIM dimer interface is abnormally hydrated with a very strange water distribution pattern--wet-core-dry-rim. The water molecules are mainly clustered compactly in a region with the residue GLU104 aside. This residue, along with several other residues in this region, is highly conserved when this region is wet. Comparing the wild type and the E104D mutant structures, no significant structural change was observed. The overall structure of the protein, including most of the large cluster of water, is maintained after the mutation. We hypothesize that, in the mutant, the water molecules in the subunit interface introduce the excessive thermolability to the protein by propagating atoms' flexibility into the core of the interface. This hypothesize was supported by the fact that atoms near the large cluster of water have a larger B factor increment than those in other interfacial regions. The hypothesize was further validated by using molecular dynamics simulations. We showed that the interfacial region near the large cluster of water was the only region that had a higher RMSD in the mutant than in the wild type.

## Competing interests

The authors declare that they have no competing interests.

## Authors' contributions

ZL, LW and JY designed the methods; ZL, QL and LZ performed experiment; YH and JL supervised the study; CKK, LW and NH participated in the data analysis; ZL, QL and JL wrote the paper; All authors read and approved the final manuscript.

## Supplementary Material

Additional file 1 **A pdf file contains Table S1, which lists the properties of the 91 homodimeric interfaces used for comparison**. Click here for file

## Declarations

Publication of this article was funded by a UTS 2013 Early Career Research Grant.

This article has been published as part of *BMC Bioinformatics *Volume 14 Supplement 16, 2013: Twelfth International Conference on Bioinformatics (InCoB2013): Bioinformatics. The full contents of the supplement are available online at http://www.biomedcentral.com/bmcbioinformatics/supplements/14/S16.
