# Supplementary material for: Structural analysis on mutation residues and interfacial water molecules for human TIM disease understanding
Source: BMC Bioinformatics. 2013 Oct 22;14(Suppl 16):S11. doi: 10.1186/1471-2105-14-S16-S11 (PMC3853089; doi:10.1186/1471-2105-14-S16-S11)
Supplement: Additional file 1 — A pdf file contains Table S1, which lists the properties of the 91 homodimeric interfaces used for comparison. [file 1471-2105-14-S16-S11-S1.PDF]

Structural analysis on mutation residues and interfacial water molecules for human TIM disease understanding

Additional file

1 Table S1

Properties of the 91 homodimeric interfaces used for comparison: resolution, number of interfacial waters, number of interfacial atoms, wetness, rWBL and gini coefficient (from column 2 to column 7).

PDB	Reso	#W	#A	wetness	rWBL	gini
12AS	2.20	12	532	0.023	0.938	0.627
1A4I	1.50	31	447	0.069	1.184	0.480
1AA7	2.08	11	319	0.034	1.062	0.611
1AD3	2.60	34	1168	0.029	0.999	0.676
1ADE	2.00	52	896	0.058	1.134	0.516
1AFW	1.80	60	812	0.074	0.932	0.559
1AJ8	1.90	46	1372	0.034	1.054	0.617
1AJS	1.60	53	1085	0.049	1.034	0.565
1AOR	2.30	16	391	0.041	0.966	0.555
1AQ6	1.95	20	672	0.030	0.896	0.660
1B3A	1.60	11	249	0.044	1.078	0.587
1B5E	1.60	31	744	0.042	1.091	0.563
1B8A	1.90	70	1379	0.051	1.043	0.576
1B9M	1.75	34	834	0.041	0.880	0.600
1BD0	1.60	101	1183	0.085	1.037	0.452
1BIS	1.95	25	496	0.050	1.029	0.558
1BJN	2.30	31	688	0.045	1.286	0.575
1BJW	1.80	24	876	0.027	1.045	0.633
1BKP	1.70	41	708	0.058	1.105	0.554
1BMD	1.90	23	521	0.044	1.184	0.570
1BRM	2.50	11	885	0.012	1.355	0.822
1BXK	1.90	27	454	0.059	0.818	0.532

1C7N	1.90	43	670	0.064	1.054	0.528
1CDC	2.00	12	934	0.013	0.938	0.798
1CG2	2.50	13	423	0.031	0.992	0.542
1CHM	1.90	85	1242	0.068	1.215	0.524
1CLI	2.50	25	753	0.033	0.911	0.580
1CNZ	1.76	13	700	0.019	0.865	0.777
1CRU	1.50	53	476	0.111	1.212	0.508
1CVU	2.40	63	936	0.067	1.039	0.474
1D0C	1.65	58	952	0.061	1.117	0.503
1DAA	1.94	22	683	0.032	1.126	0.598
1DPG	2.00	36	727	0.050	0.948	0.567
1DQS	1.80	61	663	0.092	0.832	0.399
1E9G	1.15	22	362	0.061	1.129	0.479
1ESG	1.90	20	319	0.063	0.977	0.556
1F13	2.10	76	1004	0.076	1.174	0.584
1F6Y	2.20	10	395	0.025	0.876	0.823
1G8T	1.10	21	319	0.066	0.941	0.375
1GPE	1.80	58	524	0.111	1.056	0.426
1GPU	1.86	76	1476	0.051	1.244	0.552
1H18	2.30	53	694	0.076	1.179	0.525
1HSS	2.06	17	336	0.051	1.225	0.535
1HXP	1.80	22	968	0.023	0.968	0.756
1ISA	1.80	11	303	0.036	1.164	0.658
1IVY	2.20	18	527	0.034	0.948	0.603
1JKM	1.85	55	523	0.105	1.140	0.489
1JUE	1.80	38	698	0.054	0.985	0.527
1KPE	1.80	14	548	0.026	0.701	0.705
1KQP	1.03	43	819	0.053	0.903	0.625
1M6P	1.80	22	358	0.061	1.192	0.489
1MKA	2.00	12	458	0.026	0.905	0.666
1MXR	1.42	65	1061	0.061	1.071	0.525
1OH0	1.10	16	372	0.043	0.775	0.534
1PGT	1.80	26	425	0.061	1.039	0.594
1PP2	2.50	16	382	0.042	1.199	0.652
1QFH	2.20	27	681	0.040	0.980	0.535
1QHI	1.90	26	520	0.050	1.049	0.672
1QIP	1.72	65	1151	0.056	0.989	0.545
1QKS	1.28	67	606	0.111	1.079	0.393
1QMG	1.60	74	763	0.097	1.120	0.528
1QOR	2.20	17	384	0.044	0.866	0.533
1QU7	2.60	17	1002	0.017	1.108	0.758

1R2F	2.10	25	543	0.046	0.965	0.478
1R31	2.10	47	1373	0.034	0.884	0.665
1SBY	1.10	54	828	0.065	1.007	0.602
1SG0	1.50	36	655	0.055	1.110	0.627
1SOX	1.90	39	515	0.076	1.295	0.501
1SPU	2.00	142	2302	0.062	1.169	0.564
1TC1	1.41	25	477	0.052	0.918	0.540
1U2G	2.20	26	530	0.049	1.098	0.512
1VFR	1.80	34	949	0.036	1.103	0.670
1Y6V	1.60	73	1270	0.057	1.105	0.529
1YPI	1.90	22	503	0.044	1.304	0.649
2AE2	1.90	20	428	0.047	0.871	0.530
2AL1	1.50	21	565	0.037	0.895	0.611
2GH5	1.70	69	1061	0.065	1.137	0.605
2HDH	2.20	25	468	0.053	1.122	0.638
2HHM	2.10	28	533	0.053	1.089	0.592
2JHF	1.00	31	559	0.055	1.034	0.578
2LIG	2.00	16	494	0.032	1.090	0.672
2LYN	2.07	14	295	0.047	1.103	0.549
2NAC	1.80	62	1192	0.052	0.803	0.568
2SPC	1.80	11	689	0.016	1.041	0.772
2SQC	2.00	21	295	0.071	1.032	0.451
2WE5	1.39	41	631	0.065	1.001	0.543
3DAP	2.20	18	742	0.024	0.986	0.624
3DJQ	1.53	18	538	0.033	0.857	0.657
3SDH	1.40	26	328	0.079	1.061	0.587
3TMK	2.00	12	273	0.044	0.672	0.515
5RUB	1.70	32	879	0.036	1.114	0.642
